# Alternative Types of Ambiguity and their Effects on Climate Change Regulation

**DOI:** 10.12688/openreseurope.14300.1

**Published:** 2022-01-21

**Authors:** Phoebe Koundouri, Nikitas Pittis, Panagiotis Samartzis, Nikolaos Englezos, Andreas Papandreou

**Affiliations:** 1 School of Economics, Athens University of Economics and Business, Athens, Greece; 2 Banking and Financial Management, University of Piraeus, Piraeus, Greece; 3 School of Economics, National and Kapodistrian University of Athens, Athens, Greece

**Keywords:** climate change regualtion, ambiguity, deep uncertainty, deferential ambiguity, preferential ambiguity, tail risks of environmental-policy variables

## Abstract

This paper focuses on different types of ambiguity that affect climate change regulation. In particular, we analyze the effects of the interaction among three types of agents, namely, the decision-maker (DM), the climate change experts, and the society, on the probabilistic properties of green-house gas (GHG) emissions and the formation of environmental policy. These effects are analyzed under two types of ambiguity: "deferential ambiguity" and "preferential ambiguity". Deferential ambiguity refers to the uncertainty that the experts face concerning whose forecast (scenario) the DM will defer to. Preferential ambiguity stems from the potential inability of the DM to correctly discern the society's preferences about the desired change of GHG emissions. This paper shows that the existence of deferential and preferential ambiguities have significant effects on GHG emissions regulation.

## 1 Introduction and Policy Motivation

It is often claimed that decision making on climate change is characterized by *ambiguity* (or deep uncertainty). Building on the work by Von Neumann & Morgenstern (1943) and Savage (1954), economic decision theory under uncertainty has been dominated, since the middle of the last century, by the expected utility theory and the Bayesian paradigm. A fundamental assumption in this tradition is that any source of uncertainty can be quantified in probabilistic terms, but in real-life situations, probabilities of random events are often unknown. Thus, a distinction between risk (characterizing situations in which the probabilities of an uncertain event are perfectly known) and uncertainty or ambiguity (which exists when a random event cannot be described by a probability assessment) has been increasingly used. For climate change issues it is now widely accepted that most sources of uncertainty cannot be characterized as risk, and as a consequence the expected utility theory need to be modified to include ambiguity.

Climate change policy, whether at a local, national or international scale, requires dealing with the presence of uncertainty on many dimensions. Although it is now fully recognized that the presence of these uncertainties represents an essential datum of the climate change issue, the way they should be treated and integrated in the models used to make predictions or design public policies remains an open issue. See, for example, Millner *et al.* (2013), and Lemoine & Traeger (2016) who propose numerical climate economic models under ambiguity aversion, and Chambers & Melkonyan (2017) who compare three alternative decision criteria for climate change cost-benefit analysis in the presence of uncertainty. Berger & Marinacci (2018) provide a comprehensive review of recent models of choices under uncertainty that have been proposed in the economic literature and apply them to a simple climate change decision problem.

The Special Report on Emissions Scenarios (SRES) ( Nakicenovic *et al*., 2000) generated 40 scenarios of 21st century anthropogenic greenhouse gas (GHG) emissions for the IPCC’s Third Assessment Report ( Houghton *et al*., 2001) using six different computer models and a wide range of assumptions about the values of key driving forces. Each of the 40 scenarios is based on a basic qualitative storyline that describes a future state of the world and breaks down into a number of sub-scenarios. For example, the A1 storyline (scenario family) describes a future world of very rapid economic growth, global population that peaks in mid-century and declines thereafter, and the rapid introduction of new and more efficient technologies. The A2 storyline describes a very heterogeneous world with an underlying theme of self-reliance and preservation of local identities where fertility patterns across regions converge very slowly, resulting in continuously increasing global population. In effect, we have various scenarios built under some general hypotheses for alternative future states of the world. None of these scenarios, however, includes assumptions about the behavior of the decision maker (DM) in response to the predictions to these scenarios ( *e.g*. whether DM is environmentally friendly or hostile or indifferent).

Let us have a closer look at the interaction between scenarios and the DM. The experts present the DM with the description of the scenarios and their implications on the GHG emissions. Then, the DM decides whether they will adopt a policy that is consistent with one of the scenarios. What is the probability of each possible future state of the world? Lempert *et al*. (2003 , 2006) in their editorial essay argue that in SRES ”[...] it is not possible to assign a likelihood to any of the emissions scenarios and that the associated uncertainties are best characterized by the full range of scenarios.” The experts’ weakness to assign a probability to every possible future state of the world is a central concern in this literature. Moss & Schneider (2000) have published a guideline which aims at helping experts decide the probability of each scenario: ”In addition, all authors—whether in Working Group I, II or III—should be as specific as possible throughout the report about the kinds of uncertainties affecting their conclusions and the nature of any probabilities given.”

In the case of climate change, there are two different sources of uncertainty that emerge: (i) uncertainty about which future state of the world will actually materialize, called ”ambiguity” and (ii) given that a state of the world (scenario) materializes, uncertainty regarding the model’s predictions of GHG emissions, called ”risk”. In fact, the second type of uncertainty derives from uncertainty about ”the distribution of values that a parameter, variable, or outcome may take” ( Moss & Schneider, 2000) and *not* uncertainty about the distribution of GHG emissions itself. In this paper, we focus on the first type of uncertainty ( *i.e.* ambiguity) and argue that when attempting to resolve it, one has to account for the reaction of DM. For example, a sub-scenario in SRES ( *e.g.* A1F1: very rapid economic growth, etc) assumes a ”fossil intensive” development path. Let us assume that the experts assign equal probabilities to all 40 scenarios, $\frac{1}{40}$ , and that the DM starts with some scenario and realizes that GHG emissions will be huge. This alarms the DM and makes them adopt a ”fully renewable energy” strategy, which corresponds to scenario A1T1 in SRES. By adopting this environmental friendly policy, the DM effectively increases the probability of the non-fossil energy scenario. Hence their own policy action cancels the original $\frac{1}{40}$ probability of the fossil-intensive scenario and effectively makes it smaller.

The preceding discussion highlights the fact that we cannot estimate the probability of a scenario without taking into consideration the DM’s reaction to that scenario. That is, we need to endogeneize DM’s reaction to the arrival of new information which, in the case of climate change, takes the form of alternative scenarios provided by the experts. The main argument of this paper is that the statement by SRES ”it is not possible to assign a likelihood to any of the emissions scenarios” may derive from the difficulty of endogeneizing DM’s reaction function. To the best of our knowledge there is no literature that attempts to model this endogeneity issue. Heal & Millner (2014) point to this issue: ”Even if all scientific uncertainty were resolved, we would still face major uncertainties stemming from the socioeconomic dimensions of climate change. Suppose that all scientists agreed on both a climate model and the impacts of climate change on sea level rise, fresh water availability, and so on. Even in this case they would not be able to forecast the future climate because this requires knowing future emissions. We are far from being able to forecast future emissions because they depend on whether technological change provides us with new ways of reducing GHG emissions, and the policies chosen, which are themselves difficult to forecast. Even if future emissions were known, the future would still be unknown in many important ways.” They continue using the example of sea level rise to explain their argument. In particular, they argue that even if we had an accurate forecast of the sea level for the next century, there would still remain uncertainty concerning the reaction of the society. For example, should the settlements be protected or relocated, and if relocated would these movements occur in a peaceful and organized manner or would there be strife and dislocation? Therefore, as they argue, even if a full resolution of scientific uncertainties was possible, it would not eliminate the uncertainties about the social costs of climate change.

In our model, the climate change experts provide the DM with different GHG emissions forecasts (scenarios) while the DM has to decide on which of these forecasts they will base their policy decision. The DM does not possess the epistemic status to select among the different experts’ forecasts (scenarios), which creates what we call *Deferential Ambiguity*, that is, the uncertainty the experts face concerning whose forecast the DM will defer to. A second source of ambiguity (present even in the case of a single expert) stems from the potential inability of the DM to correctly discern society’s preferences about the desired change of GHG emissions at each point in time. Hereafter, this type of ambiguity will be referred to as *Preferential Ambiguity*. An interesting question that emerges, is whether these two sources of ambiguity affect the future realization of GHG emissions. In this paper, we argue that they do and thus, it is crucial to incorporate them in a general framework that will provide insights with strong policy implications. To summarize, our work focuses on: first, characterizing the different types of ambiguity faced by the DM and the experts; and second examining their interaction and third analyzing the effects of this interaction on the probabilistic properties of GHG emissions.

## 2 Introducing the conceptual framework

Let us first describe the involved agents, namely the DM, the society and the expert(s). Consider a DM who at time *t* is about to form their system of probabilistic beliefs, that is, their subjective probability function, $P_{t}^{DM}$ , defined on a field of propositions/events Σ. The DM is assumed to be rational, which amounts to saying that (i) the DM’s subjective probability function obeys the basic probability rules for every *t*, (ii) the DM updates their probabilistic beliefs in the light of new evidence by Bayesian conditionalization ^1^ (BC) and (iii) the DM obeys the Principal Principle (see Lewis, 1980), which states that if the DM knows the objective probability (chance), *Ch*( *A*), of *A* ∈ Σ, then they set their subjective probability of A equal to the corresponding objective probability. The DM is interested in the climate variable *Y* (GHG emissions in our case), in the sense that her objective is to take the necessary policy actions to drive *Y* towards a desired level, set by the society which can be thought of as a group of agents, who have no scientific background and form *ad hoc* estimations about the desired level of the future value of GHG emissions.

An expert is defined to be the agent who knows the objective probabilities (chances), *Ch*( *A*), A ∈ Σ of the events of interest. The expert’s objective is to provide the DM with the necessary guiding information ( *i.e.* their forecast/scenarios concerning the future evolution of *Y*) that will allow the DM to implement the scenario that the society prefers. In the case of a unique expert, the DM is most likely to perceive this expert as the true bearer of the objective probabilities, which, in turn, implies that the DM will have a strong incentive (since the DM is benevolent) to defer to them at each point in time. As a result, the DM’s subjective probability distribution always coincides with the corresponding (unique) objective probability distribution. Hence, the DM always knows the true probabilities of the events of interest, which in turn implies that she always operates under an environment of ”risk” (known probabilities) rather than ”ambiguity” (unknown probabilities).

What happens though when there is more than one expert, say *n*, who disagree with each other about the chances of the events in Σ (or equivalently, when there are *n* competing scenarios for the same phenomenon)? Each of these experts has their own belief about the objective probability function on Σ (their own model). Hence, the DM is faced with *n* experts’ subjective probability functions, $P_{t}^{i},$ *i* = 1, ..., *n*, instead of one, which in turn complicates their attempts to form their subjective probability function $P_{t}^{DM}$ . How can one interpret and model these complications? A method for combining or aggregating experts’ probabilistic inputs is the so called *axiomatic method*, which is based on (i) setting a number of desirable axioms that the combined distribution should satisfy and (ii) finding the functional form that satisfies most (if not all) of these axioms. One of the most widely used functional forms is the so called linear opinion pool, according to which $P_{t}^{DM}=\sum_{i=1}^{n}w_{i}P_{t}^{i}$ , where the weights *w _i_* are non-negative and sum to one. As Clemen & Winkler (1999) remark, the weights *w _i_* may be interpreted as representing the relative quality of the *n* experts. In the case that all the experts are regarded as equivalent (by the DM) linear opinion pooling reduces to a simple arithmetic average. ^2^ Under the linear opinion pooling, there is an implicit assumption concerning the relationship among (i) the input about the phenomenon *Y* that the DM receives from the experts (ii) the DM’s actions based on this input and (iii) the actual probabilistic properties of *Y* : the DM’s actions (informed by the views of the experts) do not affect the actual probability distribution of *Y* or, put differently, the DM’s actions are exogenous to *Y*. This assumption does not seem to be realistic concerning issues of climate change.

Heal & Millner (2014) refer to the endogeneity of emissions as follows: ”Emissions uncertainty arises because anthropogenic greenhouse gas emissions drive climate change projections in all models, and future emissions pathways are unknown, as they depend on our own future policy choices.” (pp. 5). This is a case in which forecasts result in an adaptive change which in turn affects the forecasted quantity. Consequently, an expert who tries to produce a forecast for the change in *Y* between *t* and *t* + 1, Δ *Y*_*t*+1_, should integrate, in their forecast, the DM’s forecast for *Y*_*t*+1_. This feature, however, produces a two-way causality between the probabilistic views of the DM and those of the experts: the experts’ forecasts of Δ *Y*_*t*+1_ affect the DM’s forecasts of Δ *Y*_*t*+1_, but at the same time, the DM’s forecasts, being causal factors in the experts’ models for Δ *Y*_*t*+1_, affect the experts’ forecasts. As a result, a model that allows the DM to affect, *via* their actions, the determination of *Y*, must at the same time allow for a two-way causality between the DM’s and the experts’ views.

The main aim of the paper is to investigate the effects from the interaction between the DM’s and the experts’ forecasts for Δ *Y*_*t*+1_ on the actual (objective) distribution, *F*_Δ *Y*_, of the change in GHG emissions. Various forms of such interactions are analyzed, with each one generating Deferential Ambiguity ( *Def*) and/or Preferential Ambiguity ( *Pref*). *Def* is defined as follows. At each point in time, each expert *i*, *i* = 1, ..., *n*, faces the following possibilities: (a) the DM defers to his own forecasts (forecasts of expert *i*), (b) the DM defers to the forecasts of the expert *j* ≠ *i*, (c) the DM defers to a combination ( *e.g.* linear pooling) of the two experts’ forecasts and (d) the DM defers to none of the two. These possibilities raise for each of the experts the following ”specification issue”: how should the DM’s deferential attitude be introduced in each of the experts’ models? On the other hand, *Pref* stems from the potential inability of the DM to correctly discern the society’s preferences about the desired change in GHG emissions at each point in time. How should the aforementioned DM’s inability be introduced in each of the experts’ models? The way that each expert answers these questions bears different implications for the actual generation mechanism of Δ *Y*_*t*+1_.

Concerning *Pref*, assume that the society’s preference for GHG emissions formed at *t* for the value of *Y* at *t* + 1 is denoted by $Y_{t,t+1}^{*}$ , which is the sum of the current value, *Y*_*t*_, of *Y* plus a quantity *Z _t_* which represents the desired change in *Y* between *t* and *t*+1; that is, $Y_{t,t+1}^{*}$ = *Y*_*t*_ + *Z*_*t*_. This rule of dynamic determination of social preferences may be justified by assuming that in forming its desired level of *Y* for next period, the society takes into account the current level of *Y*. That is, tomorrow’s level of desired *Y* is in the neighborhood of the current level of *Y* due to physical and technological limitations. Let us further assume, that despite their best efforts, the DM fails to diagnose *Z*_*t*_, and instead believes that society’s preferences are best captured by *W*_*t*_. Alternatively, they might be able to identify *Z*_*t*_, but believe that society is currently wrong in focusing on *Z*_*t*_ and should focus on *W*_*t*_. In Tunney & Ziegler’s (2015) taxonomy, such a DM exhibits ”benevolent attitude”, in the sense that their actions are driven not by identifying ”what the society would do” but rather by ”what the society should do”. This possibility is akin to the so-called ”centralism” thesis ( Press, 1994) according to which ”ecological problems can be solved only by strong centralized control of human behavior, thus making common resource decisions by central authorities and replacing democratic rule by ’ecological mandarins’ with the ’esoteric’ knowledge and public spirit required” ( Coenen *et al*., 1998, pp5)).

Irrespective of the reasons that make the DM adopt *W*_*t*_ instead of *Z*_*t*_, the crucial question is the following. Does the expert know that the DM does not act upon *Z*_*t*_ but upon *W*_*t*_? To this end, we distinguish among three possibilities. (1) The expert (being a true expert) knows the DM’s preferential error right from the start. In such a case, the effects of the DM’s error on the actual generation process of Δ *Y*_*t*_+1 are relatively simple to analyze. The probabilistic properties of Δ *Y*_*t*_+1 do not depend on the probabilistic properties of the stochastic process { *Z _t_*}, but rather on those of { *W _t_*}. (2) The expert never realizes that the DM adopts *W*_*t*_ and erroneously believes that the DM acts on *Z*_*t*_. In this case, the actual distribution of Δ *Y*_*t*+1_ will be different than the one in the expert’s mind, which in turn implies that the expert is never the bearer of the objective chance. (3) The expert initially believes that the DM acts on *Z*_*t*_, but endorses a learning process, in the context of which they repeatedly compare the realized values of Δ *Y*_*t*+1_ with those implied by their model. Interestingly, we show that the resulting asymptotic distribution of Δ *Y*_*t*+1_ does not coincide to that in the expert’s mind, since there is a non-zero bias that survives even asymptotically, which has important policy implications.

The paper is organized as follows. Section 3, defines our basic model that examines the interactions among the involved agents, in the benchmark case where there is neither *def* nor *pref*. Section 4 introduces *pref*, which means that the expert will produce their forecasts on Δ *Y*_*t*+1_ by means of a misspecified model which integrates societal preferences instead of the DM’s actual preferences. In this section, we assume that the expert never learns about their specification error (no learning mechanism exists), which, in turn, implies that the expert ends up having a subjective probability of Δ *Y*_*t*+1_ different than the objective one (the expert is not the bearer of objective chance). Section 4.1 relaxes the no-learning assumption and derives the asymptotic distribution of Δ *Y*_*t*+1_ under Recursive Least Squares (RLS) learning. An interesting feature of this case is that the forecast error committed by the expert never goes to zero. Nevertheless, even under the aforementioned asymptotic bias, the stochastic process {Δ *Y*_*t*+1_} converges in law. Section 5 gives a brief description of how *def* can be introduced in the model, together with its potential interactions with *pref* and their combined effects on the probabilistic properties of {Δ *Y*_*t*+1_}. Section 6 summarizes the main findings and concludes the paper. The technical details of the RLS estimation method are provided in the Appendix.

## 3 The basic model

The basic model (benchmark case) is defined by the following assumptions.

**Assumptions 1**

(A1) The DM is interested in the experts’ point forecasts (conditional expectations) rather than their views about the complete distribution of Δ *Y*_*t*+1_.

(A2) The DM is a ”projectivist”, who always ( *i.e.* for each *t*) acts in such a way as to bring the actual *Y*_*t*+1_ in line with the level $Y_{t,t+1}^{*}$ designated by society at *t* as optimal for *t* + 1.

(A3) The DM acts upon *Z*_*t*_, which means that the DM’s perception about the optimal level *Y*_*t*+1_ coincides with that of the society.

(A4) There is only one expert who knows the structural form of the statistical model describing the probabilistic properties of {Δ *Y*_*t*+1_}, as well as the true values of the model’s structural parameters.

We note here that the DM’s projectivist attitude, assumed in (A2), can be fulfilled under assumption (A3). Furthermore, as a byproduct of assumptions (A3) and (A4), the expert knows that the DM acts on the basis of *Z*.

Obviously, in our benchmark case, there is neither *def* nor *pref*. As such, this case is equivalent to the basic case of Baillon *et al*. (2012) (referred to as the ”source risk”) according to which ”both agents are Bayesian and agree with each other (and everyone else). This is the common case of generally accepted objective probabilities, with no ambiguity involved” (pp.116). However, the aforementioned authors do not allow for any interactions between the DM and the expert: ”We also assume that there is no interaction between the agents themselves, or between the agents and the decision maker, so that no group process is involved.” (pp. 116). On the contrary, our model not only allows for such interactions, but makes them our central topic of research.

Let us begin with introducing some basic concepts and notation. Assume that *ε _t_* ( *Y*_*t*+1_) denotes the DM’s forecast today for the actual level of *Y* at *t* + 1, whereas $Y_{t}^{*}$ (a simplified notation for $Y_{t,t+1}^{*}$ ) stands for the level of *Y* that the society at *t* thinks of as optimal (or desired) at *t* + 1. According to (A2) of Assumptions 1, the DM is supposed to act in line with society’s preferences. This implies that whenever *ε _t_* ( *Y*_*t*+1_) > $Y_{t}^{*}$ ( *ε _t_* ( *Y*_*t*+1_) < $Y_{t}^{*}$ ), the DM acts in such a way as to produce a negative (positive) actual change Δ *Y*_*t*+1_. As far as $Y_{t}^{*}$ is concerned, we assume that it is the sum of the actual *Y _t_* and another variable *Z _t_*, with the latter representing society’s preferences at *t* for the next period’s value of *Y* , *i.e.*


$$\;Y_{t}^{*}=Y_{t}+Z_{t}\;(1)$$

If *Z _t_* > 0 ( *Z _t_* < 0), the society prefers (at *t*) a higher (lower) value of *Y*_*t*+1_ than the one that currently prevails (namely *Y _t_*). Concerning *Z _t_*, we assume that *E*( *Z _t_*) = *µ _Z_* and *V*( *Z _t_*) = $\sigma_{Z}^{2}$ . The more frequently the society’s targets change over time, the larger the value of $\sigma_{Z}^{2}$ . What are the reasons that cause the society to change the desired level of *Y* over time? One important such reason may be a change in the society’s perception/understanding about the effects of GHG emissions on social welfare. For example, if the society becomes more environmentally aware, translating in the belief that a marginal change on GHG emissions will produce higher social welfare losses, then the society will prefer a lower future level of *Y*. As McKitrick (2014) puts it: ”In a low-sensitivity model, GHG (greenhouse gases) emissions lead only to minor changes in temperature, so the socioeconomic costs associated with the emissions are minimal. In a high-sensitivity model, large temperature changes would occur, so marginal economic damages of CO _2_ emissions are larger.” (pp. 1).

Given (A3) of Assumptions 1, the DM adopts society’s target *Z _t_* and since they are supposed to act in the best interests of society, ( *ε _t_* ( *Y*_*t*+1_)− $Y_{t}^{*}$ ) enters as a causal factor in the determination of Δ *Y*_*t*+1_ with a negative coefficient. We may refer to this factor as the ”human” factor. In addition, there is a physical variable (assumed to be exogenous in the standard sense) *X*_*t*+1_, affecting Δ *Y*_*t*+1_, which may be referred to as the ”physical” factor. Bringing these two factors together results in the following equation:


$$\;\text{Δ}Y_{t+1}=Y_{t+1}-Y_{t}=\alpha (\varepsilon_{t}(Y_{t+1})-Y_{t}^{*})+\beta X_{t+1},\;(2)$$

where the structural parameters *α* and *β* are assumed to be time invariant. As regards *α* (key parameter in the ensuing analysis), we assume −1 < *α* < 0 in order to capture the DM’s socially sensitive behavior. Concerning the exogenous variable, we assume for simplicity that *X _t_* is a Gaussian IID process with zero mean, that is


$$\;X_{t}\sim NIID(0,\sigma_{X}^{2})\;(3)$$

As far as the experts are concerned, (A4) of Assumptions 1 imposes that there is only one expert who knows the structural model given by Equation (1), Equation (2) and Equation (3) together with the value of the parameter vector $\theta =\left[\alpha ,\beta ,\mu_{Z},\sigma_{Z}^{2},\sigma_{X}^{2}\right]$ . This means that the expert (at *t*) specifies in their model the same variable *Z _t_* that the DM adopts (no *pref*). Furthermore, due to (A1) of Assumptions 1, the DM always defers to the expert’s point forecast, *E _t_* (Δ *Y*_*t*+1_), and the expert is aware of this fact (no *def*). We may now provide the solution of the model in the following result.

**Proposition 2** Consider Assumptions 1 and the structural model established by Equation (1), Equation (2) and Equation (3). Then the conditional distribution of Δ *Y*_*t*+1_ is


$$\;\text{Δ}Y_{t+1}|F_{t}\sim N\left(-\frac{\alpha }{1-\alpha }Z_{t},\beta^{2}\sigma_{X}^{2}\right)\;,\;(4)$$

where *_t_* represents the information until *t*, and the corresponding unconditional distribution is


$$\text{Δ}Y_{t+1}\sim F_{basic}\left(-\frac{\alpha }{1-\alpha }\mu_{Z},\;{\left(\frac{\alpha }{1-\alpha }\right)}^{2}\sigma_{Z}^{2}+\beta^{2}\sigma_{X}^{2}\right)\;,$$

where *F _basic_* is some distribution that depends on the distribution of *Z _t_*.

**Proof.** Under (1) and (A1) of Assumptions (1), which implies that *ε _t_* ( *Y*_*t*+1_) = *E _t_* ( *Y*_*t*+1_), Equation (2) becomes


$$\text{Δ}Y_{t+1}=\alpha (E_{t}(\text{Δ}Y_{t+1})-Z_{t})+\beta X_{t+1}.$$

Taking expectations on both sides of this equation, we get *E _t_* (Δ *Y*_*t*+1_) = $-\frac{\alpha }{1-\alpha }$ *Z _t_* and therefore Δ *Y*_*t*+1_ = $-\frac{\alpha }{1-\alpha }$ *Z _t_* + *βX*_*t*+1_. The assertions of the proposition follow easily.

Below we summarize the policy implications under the benchmark case.

**Remark 3** (i) Since the coefficient $-\frac{\alpha }{1-\alpha }$ is always positive, the unconditional mean of Δ *Y*_*t*+1_ is positive (negative) whenever the mean, *µ _Z_*, of *Z* is positive (negative). This means that if the society desires, on average, a positive (negative) change in next period’s level of emissions, this desire will be translated ( *via* the DM’s actions) into an actual average positive (negative) change in emissions.

(ii) The human involvement in the generation process of *Y*_*t*+1_ always results in an increase in the variability of Δ *Y*_*t*+1_ (compared with the case for which the DM’s and society’s involvement is absent) by a factor equal to ${\left(\frac{\alpha }{1-\alpha }\right)}^{2}\sigma_{Z}^{2}$ . Put differently, even if the society (almost) always desires a lower level of next period’s emissions (that is *Z _t_* < 0), the DM’s actions to achieve this target will produce an increase in the volatility of the actual changes in emissions, compared with the cases for which the DM is inactive ( *α* = 0) or the DM is active ( *α* ≠ 0) but the society does not change its preferences over time ( *Z _t_* = 0).

(iii) Given that the society prefers a negative ( *µ _Z_* < 0) (positive *µ _Z_* > 0) change in GHG emissions, the more radical the DM ( *a* decreases) is, in terms of their policy actions towards the satisfaction of social preferences ( *e.g.* rather than regulating GHG emissions, the DM decides to adopt a fully renewable energy production model), the bigger the negative (positive) change in the actual GHG emissions Δ *Y*_*t*+1_. As far as the variance of GHG emissions is concerned, there is no ambiguity: the more radical the DM, the higher the increase in the variance of Δ *Y*_*t*+1_, irrespective of the preferences of the society.

**Figure 1.**
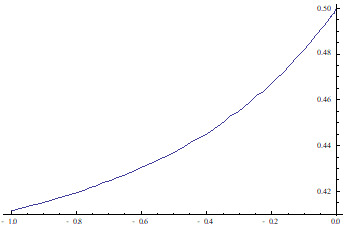
P(Δ *Y*_*t*+1_ > 0) as a function of *α*.

The aforementioned discussion bears some interesting policy implications regarding the degree of the DM’s radicalism on ”tail risks” (that is, the probability of realization of a very large change in *Y*_*t*+1_). Consider the case in which *µ _Z_* < 0, that is the case in which the society exhibits aversion to emissions. The probability of observing larger values, given on the one hand, that the mean of the distribution of Δ *Y*_*t*+1_ is shifted to the left and on the other, that the variance increases, could either increase or decrease, depending on the distribution of Δ *Y*_*t*+1_. Under the normality assumption (3), however, the aforementioned ambiguity is eliminated: the probability of observing positive changes in GHG emissions decreases as the DM becomes more radical ( *a* decreases). Figure 1 below, depicts the probability of the event *E* = {Δ *Y*_*t*+1_ > 0} as a function of *α* assuming, without loss of generality, that $\sigma_{X}^{2}$ = 1 and that *Z _t_* ~ *NIID*(−0.5, 1).

## 4 Introducing preferential ambiguity

In this section we introduce *pref* and investigate its effects on the interaction among the agents involved and the resulting change in GHG emissions. In doing so, we make the following assumptions.

**Assumptions 4** We retain the first two assumptions (A1) and (A2) of the foregoing benchmark case (see Assumptions 1) and replace (A3) and (A4) with (A3a) and (A4a), respectively, adding an additional assumption (A5) on the learning status of the expert.

(A3a) The DM acts upon *W _t_* rather than *Z _t_*.

(A4a) The unique expert knows all the features of the structural form of the model of {Δ *Y*_*t*+1_}, except of the fact that the DM employs *W _t_* instead of *Z _t_*.

(A5) The expert never learns about his specification error, generated by (A4a).

Under (A3a), the DM acts upon her own preference variable *W* rather than that of the society, *i.e.* the DM’s and society’s preferences are not aligned, and (2) becomes


$$\;\text{Δ}Y_{t+1}=\alpha (\varepsilon_{t}(\text{Δ}Y_{t+1})-W_{t})+\beta X_{t+1}\;(5)$$

The DM may or may not know *Z _t_*, while their decision to act on *W _t_* is not necessarily a strategic act towards the fulfillment of their own self-interest/agenda. As Tunney & Ziegler (2015) remark, ”surrogate decision makers may not have as their goal to match the wishes of the recipient, but instead to make what they perceive to be an optimal or benevolent decision.” (2015, pp 884).

Assumption (A4a) means that the expert will produce his forecasts of Δ *Y*_*t*+1_ by means of a misspecified model, which includes the wrong variable *Z _t_* instead of the true one, *W _t_*. Furthermore, assumption (A5) implies that the expert ends up having a subjective probability of Δ *Y*_*t*+1_ different than the objective one ( *i.e.*, the expert is not the bearer of objective chance).

**Proposition 5** Consider Assumptions 4 for the structural model given by Equation (3) and Equation (5). Then the expert’s subjective conditional distribution of Δ *Y*_*t*+1_, *i.e.*. the one perceived by the expert as true, is given by (4), the objective conditional distribution is


$$\text{Δ}Y_{t+1}|F_{t}\sim N\left(-a\left(\frac{\alpha }{1-\alpha }Z_{t}+W_{t}\right),\beta^{2}\sigma_{X}^{2}\right)\;,$$

whereas the corresponding objective unconditional distribution is


$$\text{Δ}Y_{t+1}\sim F_{pref}\left(-\alpha \left(\frac{\alpha }{1-\alpha }\mu_{Z}+\mu_{W}\right),\;{\left(\frac{\alpha^{2}}{1-\alpha }\right)}^{2}\sigma_{Z}^{2}+\alpha^{2}\sigma_{W}^{2}+\beta^{2}\sigma_{X}^{2}+2\frac{\alpha^{3}}{1-\alpha }\rho_{WZ}\sigma_{W}\sigma_{Z}\right)$$

for some distribution *F _pref_* that depends on both the distribution of *Z _t_* and *W _t_*.

**Proof.** Due to (A4a) of Assumptions 4, the expert fails to recognize the discrepancy between the DM’s and society’s preferences. Hence, they believe that (1) still holds and the law of motion of *Y*_*t*+1_ is given by Δ *Y*_*t*+1_ = *α*( *ε _t_* (Δ *Y*_*t*+1_) − *Z _t_*) + *βX*_*t*+1_. They also believe (correctly thanks to assumption (A1)) that *ε _t_* ( *Y*_*t*+1_) = *E _t_* ( *Y*_*t*+1_), *i.e.*. there is no *def*. In this case, the expert’s subjective conditional distribution is given by (4). In addition, since the DM always defers to the expert, it follows that Δ *Y*_*t*+1_ = $-\frac{\alpha^{2}}{1-\alpha }$ *Z _t_*− *αW _t_* + *βX*_*t*+1_, which implies the remaining assertions of the proposition.

Policy implications under the case of *pref* are presented below.

**Remark 6**

(i) There are cases in which the DM’s focusing on *W* instead of *Z*, that is ”deviant behavior”, results in significant shifts in the unconditional distribution of *Y*_*t*+1_, which in turn may prove beneficial for the society in the future. Specifically, if *µ _W_* < $-\frac{\alpha }{1-\alpha }$ *µ _Z_*, the DM’s actions shift the unconditional distribution to the left, thus reducing the probability of an extremely large value of Δ *Y*_*t*+1_ (tail event) in the future. This may be interpreted as the result of the DM’s benevolent behavior who acts on the basis of what the society should prefer at *t* (normative stance) rather than what the society does prefer at *t* (descriptive stance).

(ii) A similar observation can be made with respect to the unconditional variance. In particular, if *σ _Z_* > *σ _W_* and $\rho_{WZ}>\frac{(1+\alpha )\sigma_{Z}^{2}-(1-\alpha )\sigma_{W}^{2}}{2\alpha \sigma_{W}\sigma_{Z}}$ , the variance in the case with *pref*, is smaller than that in the benchmark case. In other words, under these conditions, *pref* decreases the unconditional variance, in comparison to the case of no *pref*. The human involvement in the regulation of *Y _t_* always results in an increase in the variability of Δ *Y*_*t*+1_, compared to the case of no such involvement.

(iii) The case in which the DM focuses on *W* instead of *Z* with a time-invariant policy reaction ( *i.e.* constant parameter *α*) is equivalent to the case of a DM focusing on *Z* with a time-varying policy reaction ( *i.e.* time-varying parameter *γ _t_*). To show this, it suffices to find a process { *γ _t_*} such that *α* ( *E _t_* (Δ *Y*_*t*+1_) − *W _t_*) + *βX*_*t*+1_ = *γ _t_* ( *E _t_* (Δ *Y*_*t*+1_) − *Z _t_*) + *βX*_*t*+1_. Solving for *γ _t_*, we get


$$\gamma_{t}=\frac{\alpha (E_{t}(\text{Δ}Y_{t+1})-W_{t})}{E_{t}(\text{Δ}Y_{t+1})-Z_{t}}.$$

Define $\rho_{t}=\frac{W_{t}}{Z_{t}}$ . Since *E _t_* (Δ *Y*_*t*+1_) = $-\frac{\alpha }{1-\alpha }$ *Z _t_*, the above equation becomes


$$\gamma_{t}=\frac{\alpha \left(-\frac{\alpha }{1-\alpha }Z_{t}-\rho_{t}Z_{t}\right)}{-\frac{\alpha }{1-\alpha }Z_{t}-Z_{t}}$$

and as a result, *γ _t_* = *a* ^2^ + *ρ _t_* (1 − *α*).

The above equivalence means that the DM does not have to exhibit ”deviant behavior” in order to achieve their goals. The latter may be equivalently achieved if the DM exhibits ”politically correct” behavior combined with a specific time-varying degree of policy reaction.

### 4.1 Introducing Learning

Let us now assume that the expert, utilizes the information that is being accumulated over time, to update their model by repeatedly comparing the realized values of Δ *Y*_*t*+1_ to those implied by their model.

Retaining the assumption that the expert fails to observe the discrepancy between the DM’s and society’s preferences, the only possible form of learning, is ”parameter updating”. Let the perceived (by the expert) law of motion (PLM) be


$$\;\text{Δ}Y_{t+1}=AZ_{t}+u_{t+1}\;,\;(6)$$

where *A* is the parameter that the expert tries to estimate and *u*_*t*+1_ is a Gaussian IID process with zero mean. More explicitly, in this subsection, we make the subsequent assumptions.

**Assumptions 7** We retain Assumptions 4, except for (A5) which is replaced by the new assumption:

(A5a) PLM of (6) is the reduced form model that the expert has in his mind when communicating his forecasts of Δ *Y*_*t*+1_, *i.e.*. *E _t_* (Δ *Y*_*t*+1_), to DM, where the parameter *A* is updated *via* the recursive least squares (RLS) methodology.

According to the Appendix, RLS produces an estimate ${\hat{A}}_{t}$ for each time *t*, which minimizes the mean squared error, namely *E* (Δ *Y _t_* − *E*_*t*−1_(Δ *Y _t_*)) ^2^. In fact we show that ${\hat{A}}_{t}\overset{p}{\to }A^{*}=-\frac{\alpha }{1-\alpha }\;\frac{\rho_{WZ}\sigma_{W}\sigma_{Z}+\mu_{Z}\mu_{W}}{\sigma_{Z}^{2}+\mu_{Z}^{2}}\;$ , where ” $\overset{p}{\to }$ ” signifies convergence-in-probability. Hence, asymptotically the expert’s view on *A* will settle down on A* (which is different than $-\frac{\alpha }{1-\alpha }$ ).

**Proposition 8** Consider Assumptions 7 for the structural model given by Equation (3) and Equation (5).

The asymptotic objective conditional distribution of Δ *Y*_*t*+1_ is


$$\text{Δ}Y_{t+1}|F_{t-1}\sim N\left(a(A^{*}Z_{t}-W_{t}),\beta^{2}\sigma_{X}^{2}\right)\;,$$

whereas, the corresponding objective unconditional distribution is


$$\text{Δ}Y_{t+1}\sim F_{learn}\left(a(A^{*}\mu_{Z}-\mu_{W}),\;{\left(\alpha A^{*}\right)}^{2}\sigma_{Z}^{2}+\alpha^{2}\sigma_{W}^{2}+\beta^{2}\sigma_{X}^{2}-2\alpha^{2}A^{*}\rho_{WZ}\sigma_{W}\sigma_{Z}\right)$$

for some distribution *F _learn_* that depends on the distribution of both *Z _t_* and *W _t_*.

Below we mention the policy implications under the *pref* case with learning. ^3^

**Remark 9**

(i) The asymptotic parameter A* can be decomposed in two terms: $-\frac{\alpha }{1-\alpha }$ and $\frac{\rho_{WZ}\sigma_{W}\sigma_{Z}+\mu_{Z}\mu_{W}}{\sigma_{Z}^{2}+\mu_{Z}^{2}}\;$ . The second term may be thought of as an ”adjustment factor” that captures the effects of learning. Specifically, in the no-learning case, the expert is always under the impression that the coefficient of *Z _t_* is $-\frac{\alpha }{1-\alpha }$ (4) whereas, under learning, they end up believing that this coefficient is A*. As expected, when *W _t_* ≡ *Z _t_*, the second term is equal to 1 and, A* collapses to $-\frac{\alpha }{1-\alpha }$ , that is the coefficient of the benchmark case.

(ii) For each *t*, the objective distribution of Δ *Y*_*t*+1_ does not coincide to the corresponding subjective distribution of the expert. Specifically, for each *t,* the difference between the objective conditional mean and the subjective conditional mean of Δ *Y*_*t*+1_ is given by ( *a* − 1) *A _t_Z _t_*− *αW _t_*, which, in general, is different from 0. As a result, there exists a non-zero bias at each point *t*. The important question is whether this error asymptotically vanishes. The answer to this question is, in general, negative. The corresponding difference in the asymptotic means is $\alpha \;\left(\frac{\rho_{WZ}\sigma_{W}\sigma_{Z}+\mu_{Z}\mu_{W}}{\sigma_{Z}^{2}+\mu_{Z}^{2}}\mu_{Z}-\mu_{W}\right)\;$ , which is zero iff $\rho_{WZ}=\frac{\mu_{W}\sigma_{Z}}{\mu_{Z}\sigma_{W}}\;$ . This means that in spite of the learning process, the expert never achieves a full understanding of the situation, thus committing a forecast error even asymptotically.

## 5 Introducing deferential ambiguity

Let us now introduce a second expert who (initially or permanently) disagrees with the first regarding the values of the structural parameters, *θ*, of the true model. More specifically, we assume that both experts know the true structural form of the model (including the DM’s preference variable *Z*) but take different views on the values of its parameters, with none of these experts knowing the true value of *θ.* This case corresponds to the so-called ”conflict ambiguity” in Baillon *et al*. (2012): ”For the second source of uncertainty, each agent alone fully satisfies Bayesianism, with a precise probability judgment. However, the two agents give different judgments, generating ambiguity for the decision maker aggregating their beliefs. This source of uncertainty, which is characterized by between-agent ambiguity (heterogeneous beliefs), is called conflict (C-)ambiguity in this paper.” (pp. 117).

As already mentioned, the above type of ambiguity does not exclusively affect the DM; instead because of the endogeneity of the DM’s forecasts, conflict ambiguity produces a ”boomerang effect” by injecting this ambiguity back into the process of forecast formation by the experts. Each expert does not know at each point in time whether the DM will defer to one or the other of them. Hence, each expert should account for this ambiguity by introducing it explicitly into their model for the generation of *Y*_*t*+1_. Hence, the assumptions we make in this section are the following.

**Assumptions 10**

(A1) DM adopts *Z _t_* rather than *W _t_*; *i.e.*, there is no preferential ambiguity.

(A2) There are two experts who believe that the structural model is given by Equation (1), Equation (2) and Equation (3). The two experts agree on *µ _Z_*, $\sigma_{Z}^{2}$ , $\sigma_{X}^{2}$ but disagree on *α* and *β*. Specifically, the agent i’s epistemic state is represented by $\theta_{i}=\left[\alpha_{i},\beta_{i},\mu_{Z},\sigma_{Z}^{2},\sigma_{X}^{2}\right]$ , *i* = 1, 2. Without loss of generality, assume that *α*_2_ > *α*_1_.

(A3) The objective probability that the DM at *t* defers to expert’s i point forecast, $E_{t}^{i}(\text{Δ}Y_{t+1})$ , is *p _i_*, *i* = 1, 2, where *p*_1_ + *p*_2_ = 1.

(A4) The DM combines the experts’ forecasts by means of a linear pool using *p*_1_ and *p*_2_ as weights.

(A5) Both experts know the objective deferential probabilities *p*_1_ and *p*_2_, as well as the DM’s aggregation rule.

We next have the subsequent result.

**Proposition 11** *normalfontUnder Assumptions 10 for the structural model given by Equation (1), Equation (2) and Equation (3), the conditional distribution of* Δ *Y*_*t*+1_ *is given by*


$$\text{Δ}Y_{t+1}|F_{t-1}\sim N\left(-\alpha \left(p_{1}\frac{\alpha_{1}}{1-\alpha_{1}}+p_{2}\frac{\alpha_{2}}{1-\alpha_{2}}+1\right)Z_{t},\beta^{2}\sigma_{X}^{2}\right)\;,$$

*and the unconditional distribution is*


$$\text{Δ}Y_{t+1}\sim F_{def}\left(-\alpha \left(p_{1}\frac{\alpha_{1}}{1-\alpha_{1}}+p_{2}\frac{\alpha_{2}}{1-\alpha_{2}}+1\right)\mu_{Z},\;\alpha^{2}{\left(p_{1}\frac{\alpha_{1}}{1-\alpha_{1}}+p_{2}\frac{\alpha_{2}}{1-\alpha_{2}}+1\right)}^{2}\sigma_{Z}^{2}+\beta^{2}\sigma_{X}^{2}\right)\;,$$

*where F _def_ is some distribution that depends on the distribution of Z _t_*.

**Proof.** By analogy with the proof of Proposition 2, (A1) and (A2) of Assumptions 10 imply that $E_{t}^{i}(\text{Δ}Y_{t+1})=-\frac{\alpha_{i}}{1-\alpha_{i}}Z_{t}$ , for each expert *i* = 1, 2. From (A3)-(A5) of Assumptions 10 we obtain that the actual law of motion (2) becomes $\text{Δ}Y_{t+1}=-\alpha (p_{1}\frac{\alpha_{1}}{1-\alpha_{1}}+p_{2}\frac{\alpha_{2}}{1-\alpha_{2}}+1)Z_{t}+\beta X_{t+1}$ , which yields the assertions of the proposition.

Below we summarize the policy implications under the *def* case.

**Remark 12**

(i) Since the coefficient − *α* is always positive, the unconditional mean of Δ *Y*_*t*+1_ will always be positive (negative) whenever the mean, *µ _Z_*, of *Z* is positive (negative). This means that if the society desires, on average, a positive (negative) change in next period’s level of emissions, this desire will be translated ( *via* the DM’s actions) into an actual average positive (negative) change in emissions, as in the benchmark case. This implication suggests that the existence of *def* does not affect the result that the society will end up with a change in GHG emissions that it prefers.

(ii) If both experts assume that the DM is more radical that she actually is, *i.e.* *α*_1_, *α*_2_ < *α*, the unconditional variance of the change in GHG emissions will always be smaller than in the benchmark case. In particular, in such a case, the experts’ forecasts will be suggesting smaller changes in the GHG emissions and therefore, the DM will take less radical/fewer actions, which reduces the unconditional variance of Δ *Y*_*t*+1_. If the two experts disagree on the DM’s degree of radicalness and, in particular, the first (second) expert assumes that the DM’s actions will be more (less) radical while the other believes that the DM is less (more) radical, *i.e.* *α*_1_ < *α* < *α*_2_, the unconditional variance will be larger when the probability of deference to the first expert is small enough, satisfying


$$p_{1}<\frac{\left(1-\alpha_{1})(\alpha_{2}-\alpha \right)}{\left(\alpha_{2}-\alpha_{1})(1-\alpha \right)}\;.$$

If both experts assume that the DM will be less radical than they actually are, *i.e.*. *α* < *α*_1_ < *α*_2_, then the unconditional variance of the change in GHG emissions in the *def* case of two experts, will always be larger than in the benchmark case.

## 6 Conclusions

This is the first paper, to the best of our knowledge, that introduces ambiguity that derives from the interaction among the different agents relevant in climate change regulation. The salient features of our approach are the following. (i) Ambiguity is an epistemic state which characterizes not only the DM but the scientific experts as well. We distinguish between preferential ambiguity, which is defined as the expert’s uncertainty about the DM’s preference variables and deferential ambiguity, which arises in the case of multiple experts. Deferential ambiguity may be borne by both the DM and the experts and stems from the potential difficulty of the DM to decide which of the experts should refer to. (ii) The DM’s ambiguity does not affect the formation of her prior probability function (which is the standard assumption in the ambiguity aversion literature). Instead, it affects the formation of the DM’s posterior distribution, in the sense that the DM is uncertain about the piece of information that she should condition upon. As a result, the DM’s ambiguity is compatible with probabilistic sophistication. (iii) Both types of ambiguity have significant effects on the probabilistic properties of environmental policy variables. With respect to the policy-relevant question of whether these types of ambiguity increase the probability of a ”tail event” ( *i.e.* extreme changes in GHG emissions), we show that the answer to this question depends on the probabilistic properties of the DM’s adherence to the social preferences, on the extent to which the expert(s) learns from experience, on how the DM combines experts’ information and on the pattern of interaction between preferential and deferential ambiguity.

## 7 Underlying data

No data are associated with this article.

## 8 Appendix

### 8.1 Recursive Least Squares

The least squares estimate is


$$A_{t}={\left(\sum_{s=1}^{t}Z_{s-1}^{2}\right)}^{-1}\left(\sum_{s=1}^{t}Z_{s-1}\text{Δ}Y_{s}\right)$$

More conveniently, the least squares estimates may be written in a recursive manner as


$$\;A_{t}=A_{t-1}+t^{-1}R_{t}^{-1}Z_{t-1}\left(\text{Δ}Y_{t}-A_{t-1}Z_{t-1}\right)$$

$$R_{t}=R_{t-1}+t^{-1}\left(Z_{t-1}^{2}-R_{t-1}\right)$$

where $R_{t}=t^{-1}\left(\sum_{s=1}^{t}Z_{s-1}^{2}\right)$ . The objective is to find the asymptotic value of *A _t_*, denoted by A*, and the conditions that lead to *A _t_* → A*?

The expert’s forecast of Δ *Y _t_* at time *t*−1, is given by *E*_*t*−1_(Δ *Y _t_*) = *A*_*t*−1_ *Z*_*t*−1_, which under (5) yields


$$\text{Δ}Y_{t}=\alpha \left(A_{t-1}Z_{t-1}-W_{t-1}\right)+\beta X_{t}.$$

Hence, the RLS system can be written as


$$\;\phi_{t}=\phi_{t-1}+t^{-1}R_{t}^{-1}Z_{t-1}\left(\left(\alpha -1\right)A_{t-1}Z_{t-1}-\alpha W_{t-1}+\beta X_{t}\right)$$

$$R_{t}=R_{t-1}+t^{-1}\left(z_{t-1}z_{t-1}^{\prime }-R_{t-1}\right)$$

In order to apply the standard convergence results of stochastic recursive algorithms, we need to set *S*_*t*−1_ = *R _t_*, in order for the term $R_{t}^{-1}$ in the lhs of the first equation to be a lagged variable:


$$\;\phi_{t}=\phi_{t-1}+t^{-1}S_{t-1}^{-1}Z_{t-1}\left(\left(\alpha -1\right)A_{t-1}Z_{t-1}-\alpha W_{t-1}+\beta X_{t}\right)$$

$$S_{t}=S_{t-1}+t^{-1}\left(\frac{t}{t+1}\right)\left(Z_{t}^{2}-S_{t-1}\right).$$

The associated ordinary differential equation (ODE) that governs stability of the system above is


$$\frac{d\text{Φ}}{d\tau }=h\left(\text{Φ}\right)=\underset{t\to \infty }{\lim}E\left(Q\left(t,\text{Φ},zt\right)\right)$$

where Φ = ( *A, S*) ^*'*^, *z _t_* = ( *Z*_*t*−1_, *W*_*t*−1_, *X _t_*) and *E* denotes the expectation of *Q*( *t,* Φ, *z _t_*) taken over the invariant distribution of *z _t_*, for fixed Φ. *Q* ( *t*, Φ, *z _t_*) is derived by the RLS system and is defined as


$$Q\left(t,\text{Φ},z_{t}\right)=\left(\begin{array}{c} S^{-1}Z_{t-1}\left(\left(\alpha -1\right)AZ_{t-1}-\alpha W_{t-1}+\beta X_{t}\right)\\ \left(\frac{t}{t+1}\right)\left(Z_{t}^{2}-S\right) \end{array}\right)$$

It follows that


$$\begin{array}{l} h_{\theta }\left(\text{Φ}\right)\;=\;\underset{t\to \infty }{lim}E\left(S^{-1}Z_{t-1}\left(\left(\alpha -1\right)AZ_{t-1}-\alpha W_{t-1}+\beta X_{t}\right)\right)\\ h_{S}\left(\text{Φ}\right)\;=\;\underset{t\to \infty }{lim}\left(\frac{t}{t\;+\;1}\right)E\left(Z_{t}^{2}-S\right)=\left(\sigma_{Z}^{2}+\mu_{Z}^{2}\right)-S \end{array}$$

The second relationship gives $S\to \left(\sigma_{Z}^{2}+\mu_{Z}^{2}\right)$ , and therefore,


$$\begin{array}{l} h_{\theta }\left(\text{Φ}\right)\;=\;\underset{t\to \infty }{lim}E\left({\left(\sigma_{Z}^{2}+\mu_{Z}^{2}\right)}^{-1}Z_{t-1}\left(\left(\alpha -1\right)AZ_{t-1}-\alpha W_{t-1}+\beta X_{t}\right)\right)=\\ \;=\left(\alpha -1\right)A-\alpha {\left(\sigma_{Z}^{2}+\mu_{Z}^{2}\right)}^{-1}\left(\rho_{WZ}\sigma_{W}\sigma_{Z}+\mu_{Z}\mu_{W}\right) \end{array}\;(7)$$

The ODE ( 7) gives the system


$$\dot{A}\;=\;\left(\alpha -1\right)A-\alpha {\left(\sigma_{Z}^{2}+\mu_{Z}^{2}\right)}^{-1}\left(\rho_{WZ}\sigma_{W}\sigma_{Z}+\mu_{Z}\mu_{W}\right)$$

whose solution is given by


$$A^{*}=-\frac{\alpha }{1-\alpha }\frac{\rho_{WZ}\sigma_{W}\sigma_{Z}+\mu_{Z}\mu_{W}}{\sigma_{Z}^{2}+\mu_{Z}^{2}}$$

The convergence to A* is convergence in probability, i.e. ∀ *ε* > 0, lim _*t*→∞_ *P* (| *A _t_*− A*| ≥ *ε*) = 0. The E-stability amounts to the condition *α* < 1, which holds by assumption. Therefore, with probability 1, the system will converge to the equilibrium, irrespective of the initial estimations *A*_0_.

### 8.2 Comparison of Variances in Section 3 & Section 4

The unconditional variance for all the above cases can be written in a general form as


$$\alpha^{2}\left[{\left(\frac{\alpha }{1-\alpha }R\sigma_{Z}+\sigma_{W}\right)}^{2}-2\frac{\alpha }{1-\alpha }R\left(1-\rho_{WZ}\right)\sigma_{Z}\sigma_{W}\right]+\beta^{2}\sigma_{X}^{2}$$

To arrive at the first case, we have that *R* = *ρ _WZ_* = 1, *σ _W_* = *σ _Z_*. For the second case, we need *R* = 1 and the third $R=\frac{\rho_{WZ}\sigma_{W}\sigma_{Z}+\mu_{Z}\mu_{W}}{\sigma_{Z}^{2}+\mu_{Z}^{2}}$ . Note that the unconditional variance is increasing (decreasing) in *R* if $R>\left(<\right)-\frac{1-\alpha }{\alpha }\frac{\sigma_{W}}{\sigma_{Z}}\rho_{WZ}$ .
