# Supplementary material for: Alternative Types of Ambiguity and their Effects on Climate Change Regulation
Source: Open Res Eur. 2022 Jan 21;2:9. [Version 1] doi: 10.12688/openreseurope.14300.1 (PMC10445894; doi:10.12688/openreseurope.14300.1)
Supplement: Peer review history

# Peer review: Reviewer response for version 1

**DOI:** 10.21956/openreseurope.15428.r31497

**Published:** 2023-06-16

**Authors:** Marcela Brugnach

**Affiliations:** 1 BC3 Basque Climate Change Center, Leioa, Spain

The research presented in this paper examines the effects of deferential and preferential ambiguity in GHG emissions regulation. A subject that is currently very important in the context of climate change. The article is well written and structured, and it suggests a step forward in our knowledge of the implications of ambiguity in decision-making. I strongly suggest that it be indexed, but before I would like the authors to address the following point:

 Issues of ambiguity are addressed here from the standpoint of economic decision theory, leaving aside more interpretative frameworks of uncertainty that have long included ambiguity as a different kind of uncertainty. While the authors concluded that this is the first work to address the ambiguity that arises from the interaction of the various agents involved in climate change regulation, I propose that they broaden their discussion and conclusions to address the already existing, and extensive, works on the subject, even if from a very different perspective. In addition to acknowledging earlier work, I hope this will spark a really interesting discussion regarding the methodological and philosophical differences between different approaches.

If applicable, is the statistical analysis and its interpretation appropriate?

Yes

Is the study design appropriate and is the work technically sound?

Yes

Is the work clearly and accurately presented and does it engage with the current literature?

Partly

Are the conclusions drawn adequately supported by the results?

Partly

Are sufficient details of methods and analysis provided to allow replication by others?

Yes

Are all the source data and materials underlying the results available?

Yes

Reviewer Expertise:

Collective decision-making under uncertainty (incl. ambiguity)

I confirm that I have read this submission and believe that I have an appropriate level of expertise to confirm that it is of an acceptable scientific standard, however I have significant reservations, as outlined above.

# Peer review: Reviewer response for version 1

**DOI:** 10.21956/openreseurope.15428.r31891

**Published:** 2023-06-12

**Authors:** Laurent Li

**Affiliations:** 1 Laboratoire de Météorologie Dynamique, CNRS, Sorbonne Université, Paris, France

This manuscript describes a framework potentially useful for climate change policy making. The framework involves three stakeholders on the material: the Expert on anthropogenic climate change, the Society or the public and the Decision-maker (DM) for relevant regulation. It deals with the concept of “ambiguity”, to be differentiated from the “risk”. The latter represents uncertainty which can be described in probabilistic terms, while the former represents uncertainty which can’t be easily expressed by a probability. The authors introduced two kinds of ambiguity (deferential ambiguity and preferential ambiguity) for the DM in elaborating climate change regulation. The deferential ambiguity is related to the appreciation of the DM for the uncertain expertise provided by the Expert of climate change, while the preferential ambiguity is related to the preference of the Society. I am not at all a specialist of theories and practices of policy decision making, but I find this framework interesting, and potentially useful for climate change policy making. I have a few comments that might be helpful for the improvement of the manuscript:

1. It is not easy for a general reader to fully understand the proposed framework, neither its practical implementation. It would be much appreciable if the authors can make efforts in showing diagrams, tables, schematics, flowing charts, figures, and so on.
2. I would strongly encourage the authors to fully illustrate their framework and main concepts by using more examples, even by constructing a toy model. It would be an effort useful and very much appreciated if examples can be illustrated with climate change issues.
3. Without relevant application to climate change issues, the section of conclusions seems a little vague and not precise. It is stated that “Ambiguity is an epistemic state which characterizes not only the DM but the scientific experts as well”. I thought the paper was on the deferential and preferential ambiguities for the DM, not for the scientific expertise? I am much confused. By the way, in a few places, the manuscript uses “she” or “her” for the DM. Does that mean the DM should be a female person? I would use more neutral terms “she/he” and “her/his”.
4. The authors use IPCC’s SRES (Special Report on Emissions Scenarios) to introduce and illustrate the general steps and potential difficulties for the decision making on climate change policy. However, SRES is a little old to describe future emission of greenhouse gases, including its different narratives such as A1, A2, A1F1 mentioned in the paper. IPCC's concept has been moved to RCP (Representative Concentration Pathways), and then to SSP (Shared Socioeconomic Pathways) in its recent assessment reports.
5. Certainly, it is an issue that we don't know if the DM would adopt a policy that is consistent with an emission scenario. But how about the adhesion or compliance of the public or the Society? Should we place this unknown at the same level of the DM's ambiguity?

If applicable, is the statistical analysis and its interpretation appropriate?

Yes

Is the study design appropriate and is the work technically sound?

Yes

Is the work clearly and accurately presented and does it engage with the current literature?

Yes

Are the conclusions drawn adequately supported by the results?

Partly

Are sufficient details of methods and analysis provided to allow replication by others?

Yes

Are all the source data and materials underlying the results available?

Yes

Reviewer Expertise:

I am a climate modeler for the physical part of the climate system. My research is mainly on the assessment of climate change impacts

I confirm that I have read this submission and believe that I have an appropriate level of expertise to confirm that it is of an acceptable scientific standard, however I have significant reservations, as outlined above.

# Peer review: Reviewer response for version 1

**DOI:** 10.21956/openreseurope.15428.r29238

**Published:** 2022-08-11

**Authors:** John Beirne

**Affiliations:** 1 Asian Development Bank Institute, Tokyo, Japan

This paper examines the role of alternative types of ambiguity on climate change regulation. In particular, two forms of ambiguity are defined and examined: deferential ambiguity and preferential ambiguity. For the former, uncertainty pertains to that encountered by climate change experts in ascertaining which climate change scenario is adopted by the decision maker. The latter form relates to the decision makers’ uncertainty in accurately determining the preferences of society on climate change. Defining these terms is of key importance, not least given the lack of consensus on the term “ambiguity” itself, although there is generally more agreement in the behavioral economics literature (Arend, 2020). ^1^ The paper is motivated on the basis that standard models of expected utility theory and optimization fail to appropriately account for different types of ambiguity (where the probabilities of outcomes are unknown) and that issues around climate change fall into the category of policy concerns whose assessment would benefit from modified models of expected utility.

 The paper recognizes that uncertainty on climate change induced outcomes has been dealt with in the literature in various ways, such as through multi-scenario and cost-benefit analyses. A recent paper by Berger and Marinacci (2020) provides a framework for incorporating uncertainty into decision models on optimal climate change mitigation policies. ^2^ The current paper goes further by accounting for the behavior of the decision maker under two alternative ambiguity scenarios. Moreover, as deferential ambiguity and preferential ambiguity affect the future level of greenhouse gas emissions, the authors rationalize that both should be incorporated into the modelling framework for policy choices on climate change. The paper describes intuitively the baseline model, which is then augmented with preferential and deferential ambiguity components, also explaining how the interaction of these ambiguities affects the probabilistic properties of greenhouse gas emissions. The theoretical framework is clearly and methodically described, and also could extend very well to the prevailing wider literature on modelling other policy decisions under ambiguity.

 The main contribution of the paper is on introducing in a coherent and integrated theoretical framework two forms of ambiguity that affect policy outcomes applied to climate change. The details provided by the authors should be sufficient to enable a replication of the framework, and the conclusions drawn support the theory presented. Moreover, the framework appears to be technically sound and internally consistent. That said, the findings are necessarily underpinned by theoretical assumptions on, for example, societal preferences on greenhouse gas emissions or experts’ perceptions of the behavior of the decision maker. It follows that adjustments to underlying assumptions could lead to alternative results and sensitivity exposures. The general applicability of the results to the current debate on climate change regulation should be nuanced by the fact that conclusions may be affected by adjustments in model parameters and theoretical assumptions imposed. The paper would benefit from a discussion of applications of the theoretical framework derived and its implications for policy makers. This may be achieved by illustrating the theoretical inferences with a basic numerical or simulation exercise. Such an exercise may also add clarity to the authors’ question of whether the ambiguities considered in the paper are positively correlated with the probability of a tail-risk occurrence, such as an extreme rise in greenhouse gas emissions. The answer provided indicates that such an outcome is dependent on a number of interrelated factors, although there is scope to elaborate and explain further with simulated scenarios.

 While the paper provides an important contribution to enhancing our understanding of the complex nature of interactions amongst agents in the presence of alternative forms of ambiguity, a question remains as to which type of ambiguity has the greatest relative effect on the posterior distribution of the decision maker and what the appropriate weights should be. In addition, it would be useful to ascertain which type of ambiguity drives regulation on greenhouse gas emissions. The paper makes a theoretical case for both factors, but additional sensitivity analysis would be beneficial. Related to this, the authors may explain further why the approach undertaken is necessarily theoretical, highlighting some of the factors that may impede real-world application.

 Building on this, further numerical simulations would demonstrate whether the optimal policy response would equate to strict or gradual abatement measures on greenhouse gas emissions. Moreover, allowing for time variation in the incidence of deferential and preferential ambiguity would bring additional relevance of the generic approach as the theoretical underpinning for policy choices on climate change regulation. This could include extensions to account for non-linearities in adaptive learning, particularly from a baseline of status quo deference, and deriving welfare-enhancing optimality in policy decisions.

**References**

 Arend, R. (2020). “Strategic Decision-making under Ambiguity: A New Problem Space and a Proposed Optimization Approach”, *Business Research *13: 1231–1251.

 Berger, L. and Marinacci, M. (2020). “Model Uncertainty in Climate Change Economics: A Review and Proposed Framework for Future Research”, *Environmental and Resource Economics* 77: 475–501.

If applicable, is the statistical analysis and its interpretation appropriate?

Not applicable

Is the study design appropriate and is the work technically sound?

Yes

Is the work clearly and accurately presented and does it engage with the current literature?

Yes

Are the conclusions drawn adequately supported by the results?

Partly

Are sufficient details of methods and analysis provided to allow replication by others?

Yes

Are all the source data and materials underlying the results available?

Yes

Reviewer Expertise:

International finance; financial economics; macroeconomics

I confirm that I have read this submission and believe that I have an appropriate level of expertise to confirm that it is of an acceptable scientific standard.

## References

[1] : Strategic decision-making under ambiguity: a new problem space and a proposed optimization approach. *Business Research* .2020;13(3) : 10.1007/s40685-020-00129-7 1231-1251 10.1007/s40685-020-00129-7

[2] : Model Uncertainty in Climate Change Economics: A Review and Proposed Framework for Future Research. *Environmental and Resource Economics* .2020;77(3) : 10.1007/s10640-020-00503-3 475-501 10.1007/s10640-020-00503-3
